# Group 2 Innate Lymphoid Cell Production of IL-5 Is Regulated by NKT Cells during Influenza Virus Infection

**DOI:** 10.1371/journal.ppat.1003615

**Published:** 2013-09-19

**Authors:** Stacey Ann Gorski, Young S. Hahn, Thomas J. Braciale

**Affiliations:** 1 Beirne B. Carter Center for Immunology Research, University of Virginia, Charlottesville, Virginia, United States of America; 2 Department of Microbiology, University of Virginia, Charlottesville, Virginia, United States of America; 3 Department of Pathology, University of Virginia, Charlottesville, Virginia, United States of America; University of Pennsylvania, United States of America

## Abstract

Respiratory virus infections, such as influenza, typically induce a robust type I (pro-inflammatory cytokine) immune response, however, the production of type 2 cytokines has been observed. Type 2 cytokine production during respiratory virus infection is linked to asthma exacerbation; however, type 2 cytokines may also be tissue protective. Interleukin (IL)-5 is a prototypical type 2 cytokine that is essential for eosinophil maturation and egress out of the bone marrow. However, little is known about the cellular source and underlying cellular and molecular basis for the regulation of IL-5 production during respiratory virus infection. Using a mouse model of influenza virus infection, we found a robust transient release of IL-5 into infected airways along with a significant and progressive accumulation of eosinophils into the lungs, particularly during the recovery phase of infection, i.e. following virus clearance. The cellular source of the IL-5 was group 2 innate lymphoid cells (ILC2) infiltrating the infected lungs. Interestingly, the progressive accumulation of eosinophils following virus clearance is reflected in the rapid expansion of c-kit^+^ IL-5 producing ILC2. We further demonstrate that the enhanced capacity for IL-5 production by ILC2 during recovery is concomitant with the enhanced expression of the IL-33 receptor subunit, ST2, by ILC2. Lastly, we show that NKT cells, as well as alveolar macrophages (AM), are endogenous sources of IL-33 that enhance IL-5 production from ILC2. Collectively, these results reveal that c-kit^+^ ILC2 interaction with IL-33 producing NKT and AM leads to abundant production of IL-5 by ILC2 and accounts for the accumulation of eosinophils observed during the recovery phase of influenza infection.

## Author Summary

IL-5 is a cytokine that is typically associated with parasitic infections and allergic reactions. The primary role of IL-5 is thought to be for the development and maturation of an innate immune cell type, the eosinophil, which is also a culprit in allergic diseases such as asthma. During respiratory virus infection, such as influenza infection, IL-5 and eosinophils are not thought to play a major role in host defense. Here we show that IL-5 is produced in response to influenza infection and results in the progressive accumulation of eosinophils in the lung. We show that a newly discovered cell type, the group 2 innate lymphoid cell (ILC2), is responsible for IL-5 production during influenza infection and that the capacity of ILC2 to make IL-5 is greatly increased following virus clearance, i.e. during the recovery phase. The production of IL-5 by ILC2 is in part regulated by NKT cells and IL-33 produced by this cell type during the recovery phase of influenza infection.

## Introduction

Type 2 immune responses are induced by parasitic and helminth infection and are characterized by the production of prototypical cytokines such as IL-4, -5, and -13 [1]. IL-5 is one of the major type 2 cytokines that is essential for eosinophil survival (in humans) as well as B1-B cell development in mice [2]. Although beneficial during parasitic or helminth infection, IL-5 may have a detrimental role in the development and severity of asthma and allergic diseases. Because of its essential role in eosinophil generation in the bone marrow and eosinophil egress out of the bone marrow, local production of IL-5 in the lungs during asthma exacerbation can result in pulmonary eosinophilia which can in turn enhance airway smooth muscle contraction and cause excess mucus production [3], [4].

Although viral infections are generally considered to elicit classic type 1 immune responses, features of the type 2 response are frequently present particularly in individuals with pre-existing allergic diseases e.g. asthma [5], [6]. The presence of type 2 cytokines during respiratory virus infection has been linked to asthma exacerbation; however, there is an emerging view that the type 2 responses might also play a tissue protective role [7], [8].

Group 2 innate lymphoid cells (ILC2) are innate immune lymphocyte-like cells that are capable of producing large amounts of IL-5 and IL-13 when stimulated by IL-25 and IL-33, two cytokines associated with the type 2 response [9]. ILC2 were first identified in fat associated lymphoid clusters and subsequently other groups identified similar cells in the gut, spleen and lung [10]–[12]. ILC2 present in the lung of mice infected with influenza A virus (IAV) have been reported to produce abundant IL-13 during infection, which may contribute to airway hyperreactivity observed during experimental infection with respiratory viruses such as IAV [13]. However, Monticelli et al. found that ILC2 are also capable of producing the epidermal growth factor family member amphiregulin, and this is essential to proper repair of the epithelial barrier following IAV infection [14].

In this report we demonstrate that during IAV infection there is abundant production of IL-5 in the infected respiratory tract, which stimulates the progressive recruitment and accumulation of eosinophils in the infected lungs, particularly late in infection (i.e. after infectious virus clearance) during the recovery phase. This IL-5 is primarily the product of a small number of ILC2 recruited to the IAV infected lungs. Interestingly, while ILC2 produce IL-5 during the acute phase (i.e. 5–7 d.p.i., which corresponds to IAV-specific adaptive immune cell influx into the lungs), both ILC2 numbers and their ability to produce IL-5 increases dramatically following infectious virus clearance during the recovery phase (8–10 d.p.i. and beyond). This increase in IL-5 production by ILC2 is in part stimulated by IL-33 produced by both alveolar macrophages (AM) and, unexpectedly, NKT cells infiltrating the IAV infected lungs. The significance of these findings is discussed.

## Results

### IL-5 is produced in the lungs during IAV infection

**Figure 1:**
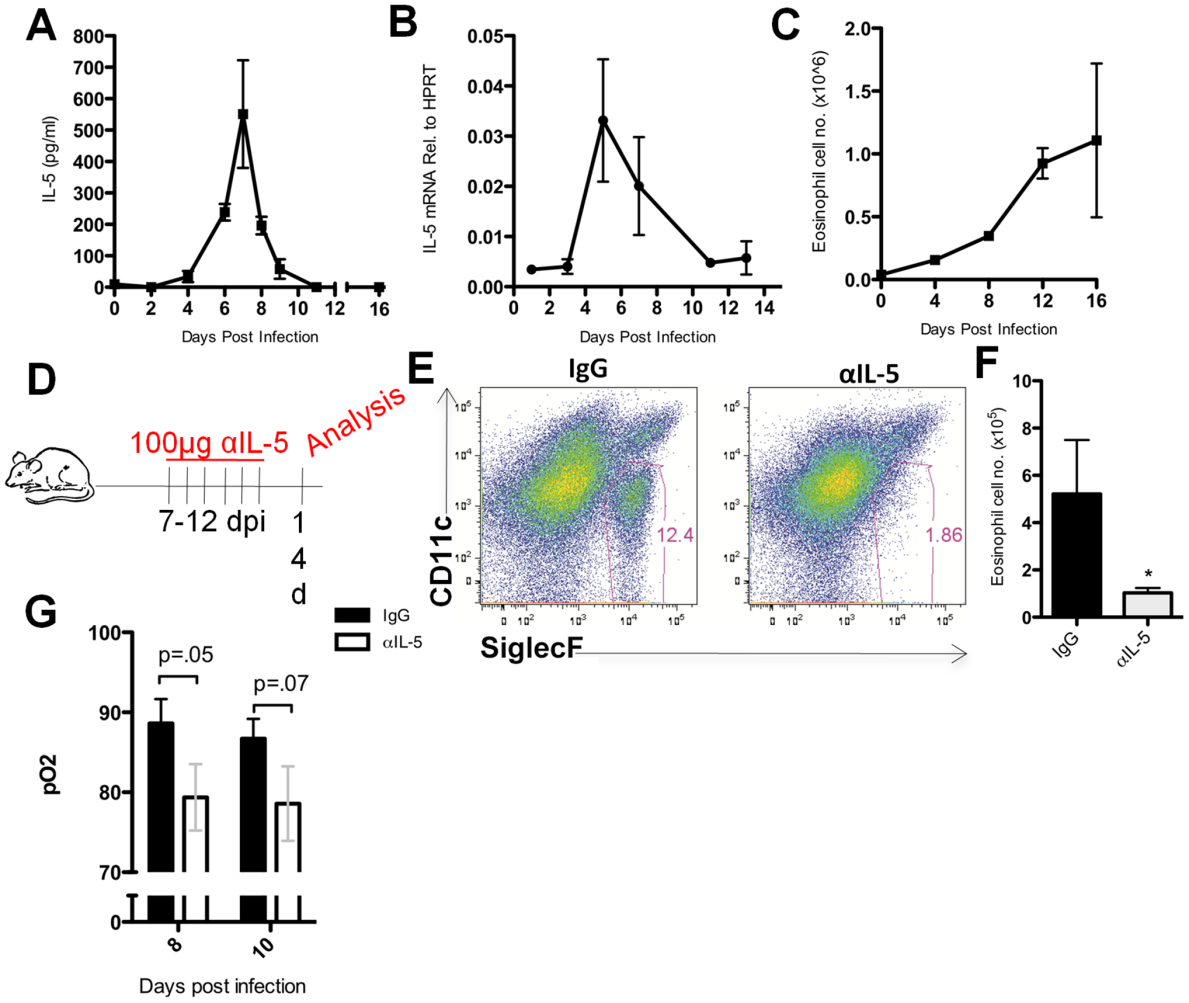
IL-5 is produced in the influenza-infected lung and results in eosinophil accumulation in the lung. (A) BAL fluid from C57BL/6 mice was collected and IL-5 levels were measured via ELISA. (B) cDNA was derived from whole lung homogenate and analyzed for IL-5 transcript as described in material and methods. (C) Total eosinophils present in the lungs as identified by flow cytometry. (D) BALB/c mice were given 100 µg of neutralizing anti-IL-5 antibody (αIL-5) i.p. daily from 7–12 d.p.i. and lung cell suspensions analyzed at 14 d.p.i.. (E) Eosinophils were identified in the lung as CD45^+^SiglecF^+^CD11c^lo^. Plots represent analysis of lung cell suspensions first gated on CD45^+^ cells. (F) Total number of eosinophils present in the lung at 14 d.p.i. following αIL-5 treatment. (G) Oxygen saturation levels (pO_2_) of mice given daily i.p. injections of αIL-5 or IgG beginning at 7 d.p.i. Bars represent SEM of a representative experiment repeated at least three times. N = 3–5 mice per experiment. *p<.05.

As part of a survey of cytokines produced in the respiratory tract following experimental influenza A virus (IAV) infection, we confirmed earlier reports [15]–[17] that IL-5 is released into the bronchoalveolar lavage (BAL) of IAV infected mice, in this instance mice infected with the mouse adapted IAV strain A/PR/8/34. IL-5 protein was first reproducibly detected in the BAL at 4 days post infection (d.p.i.), released at maximum levels in the BAL at 7 d.p.i. and detectable IL-5 in the BAL fluid subsequently declined over the next several days, concomitant with the clearance of infectious virus (Figure 1A). In contrast to IL-5 protein expression, IL-5 gene expression (mRNA levels) in homogenates of infected lungs, while paralleling the kinetics of release of this cytokine into the BAL fluid early during infection (Figure 1B), IL-5 transcripts remained readily detectable beyond 8 d.p.i. in spite of the absence of detectable IL-5 protein in the BAL at this time. (Figure 1B). By 8 d.p.i. there is a massive influx of CD45^+^ immune/inflammatory cells, including both adaptive immune cells (e.g. T and B cells) as well as myeloid lineage inflammatory cells [18], thus the apparent reduction in IL-5 transcripts detectable in lung homogenates late in infection is likely an underestimate of the actual transcript level. This would particularly be true if the IL-5 gene is expressed by a limited number of cells and the IL-5 mRNA in lung homogenate is diluted by RNA extracted from the CD45^+^ (IL-5 mRNA^−^) inflammatory cells accumulating within the lungs. Accordingly, the markedly diminished level of detectable IL-5 protein during the latter days following infection could reflect consumption of the cytokine by one or more cell types infiltrating the infected lungs.

IL-5 is the primary cytokine involved in the generation and maturation of eosinophils in the bone marrow and for the egress of eosinophils to sites of infection [19] e.g. the IAV infected lungs. Indeed, when we examined the kinetics of eosinophil accumulation in the infected lungs, we found that eosinophils accumulated progressively during the course of infection with the most pronounced accumulation of this cell type during the recovery phase of infection i.e. from 8 d.p.i.–16 d.p.i. (Figure 1C). In order to establish that eosinophil accumulation in the lungs during the recovery phase was dependent on IL-5, infected mice were treated with neutralizing anti-IL-5 antibody (αIL-5) daily between 7 and 12 d.p.i. (Figure 1D). IL-5 neutralization resulted in a substantial reduction of eosinophils in the lung at 14 d.p.i. (Figure 1E and 1F). *In vivo* neutralization of IL-5 resulted in reduced O_2_ saturation at days 8 and 10 p.i., however, this did not reach statistical significance (Figure 1G). It is noteworthy that if α-IL-5 administration were limited to the period from 7 through 10 d.p.i. and lung eosinophil numbers evaluated 4 days later, i.e. at 14 d.p.i., eosinophil numbers in the lungs were comparable to control IgG -treated animals (Figure S1A and S1B). Taken together these data suggest that IL-5 not only continues to be produced in the influenza-infected lung during the recovery phase, but also is responsible for the progressive accumulation of eosinophils in the lungs. Therefore, our failure to demonstrate detectable levels of IL-5 protein in the BAL fluid at 8 d.p.i. and beyond most likely reflects the consumption of the cytokine by IL-5 receptor expressing cells, i.e. eosinophils.

### Group 2 innate lymphoid cells (ILC2) are the primary producers of IL-5 during IAV infection

We next sought to identify the cellular source of IL-5 in the infected lungs. Since the kinetics of IL-5 release into the BAL fluid (Figure 1A) was comparable to the kinetics of many pro-inflammatory cytokines (e.g. IFN-g) that are released into the IAV infected lungs by responding adaptive immune IAV-specific CD8^+^ and CD4^+^ T-cells [20] we considered that one or more adaptive immune cell type was a likely source of the IL-5. To explore this possibility, we interrogated various cell types in the IAV infected lungs for expression of IL-5 transcripts. To this end, we first separated total lung cell suspensions from mice at 5 d.p.i. into Ly6G^+^ (primarily neutrophils), Thy1.2^+^ (lymphocytes), DCs (CD11c+MHC II^hi^) and CD45^−^ (epithelial/stromal origin). We observed that IL-5 gene expression was primarily associated with Thy1.2^+^ cells, with a minimal amount being detected in the CD45^−^ fraction (Figure S2A). We then isolated by cell sorting of Thy1.2^+^ lung cells, the CD8^+^ T cell, CD4^+^ T cell, NKT cell, and NK cell fractions along with the residual lineage^−^ Thy1.2^+^ cells. Somewhat unexpectedly, IL-5 mRNA was detected primarily in the Thy1.2^+^ Lin^−^ cell fraction (Figure S2B). Consistent with these findings, depletion of CD4, CD8 or NK cells had minimal impact on BAL IL-5 levels (Figure S2C). In addition, mice deficient in gamma delta T cells (TCRγδ-/-), another potential Thy1.2^+^ cell type, did not significantly alter BAL IL-5 levels (Figure S2D). Jα18-/- mice, which are devoid of type I NKT cells, did have consistently lower BAL IL-5, however, due to the absence of IL-5 transcripts in the isolated NKT cell population, NKT cells were not considered a likely source of IL-5. These findings therefore suggested that the primary source of IL-5 in the IAV infected lungs was the recently described Thy1^+^ Lin^−^ cell population, the group 2 innate lymphoid cell (ILC2).

**Figure 2:**
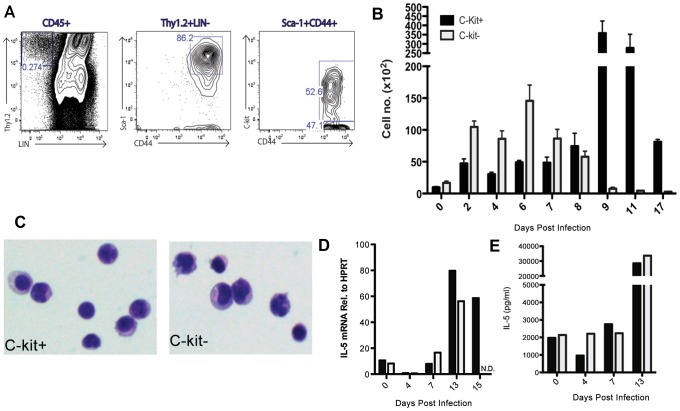
Group 2 innate lymphoid cells (ILC2) expand and are more potent producers of IL-5 during the resolution phase of infection. (A) Representative FACS plots identifying ILC2 in C57BL/6 mice. LIN antibody cocktail included: CD3, CD4, CD5, CD8, CD11b, Gr-1, CD19, B220, DX5 (or NK1.1) and TCRδ. (B) Absolute number of both c-kit^+^ and c-kit^−^ ILC2 following infection. (C) c-kit^+^ and c-kit-ILC2 were FACS sorted from the lung at 7 d.p.i. and stained with hematoxylin and eosin. (D–E) c-kit^+^ and c-kit-ILC2 were FACS sorted from the lung at indicated times and (D) directly analyzed for IL-5 transcript or (E) cultured for three days in the presence PMA/ionomycin. (E) Supernatants were collected and IL-5 levels analyzed via ELISA. Data is representative of two experiments with ≥3 mice per group (A–C) or pooled from 15–20 mice (D–E). Bars = +/− SEM.

ILC2 were first identified in fat associated lymphoid clusters and have since been detected in the spleen, mesenteric lymph node, as well as lungs [9]–[12] including IAV infected lungs [13], [14]. These cells produce extremely high levels of the type 2 cytokines IL-5 and IL-13 (on a per cell basis) when stimulated with IL-25 or IL-33. ILC2 have generally been described as LIN^−^Thy1.2^+^ CD44^+^Sca-1^+^ and either c-kit^+^ [10] or c-kit^−^ [12] depending on the tissue source or experimental conditions. We detected both c-kit^+^ and c-kit^−^ ILC2 in the IAV infected lungs (Figure 2A). While both c-kit^+^ and c-kit^−^ ILC2 accumulate in the lungs following IAV infection, the c-kit^−^ ILC2 subset predominates during the acute phase of infection (2–7 d.p.i.) with the c-kit^+^ ILC2 rapidly accumulating in increasingly large numbers during the recovery phase i.e. after infectious virus clearance at 8–9 d.p.i. and beyond (Figure 2B). Both ILC2 subsets appeared to be morphologically similar (Figure 2C) and expressed similar levels of various surface markers (Figure S3). In support of previously published reports, both c-kit^+^ and c-kit^−^ ILC2 expressed CD25, CD127 and the IL-33 receptor subunit, ST2 [21], as well as MHC II and CD69. We detected little to no FLT3, a hematopoietic growth factor receptor that is in the same family as c-kit and serves a similar function [22]. Each subset was also equally capable of expressing amphiregulin (Figure S4), as this epidermal growth factor family member was recently shown to be critical for epithelial barrier protection during IAV infection [14], [23].

Following purification by cell sorting, both ILC2 subsets expressed comparable levels of IL-5 transcripts when analyzed directly *ex vivo* (Figure 2D) as well as comparable IL-5 secretion following *in vitro* stimulation of equivalent numbers of sorted cells with PMA/ionomycin (Figure 2E) suggesting functional as well as morphological similarity of the two ILC2 subsets. To determine if the rapid accumulation of c-kit^+^ ILC2 was the result of local proliferation of c-kit^+^ ILC2 in the respiratory tract, we evaluated ongoing proliferation of this cell type by Ki67 expression at 10 d.p.i. (Figure S5A) and BrdU uptake at 7 d.p.i. (Figure S5B). We were unable to detect significant proliferation of these cells by either criteria suggesting that the c-kit^+^ ILC2 accumulating in the IAV infected lungs were most likely being recruited from the bone marrow.

**Figure 3:**
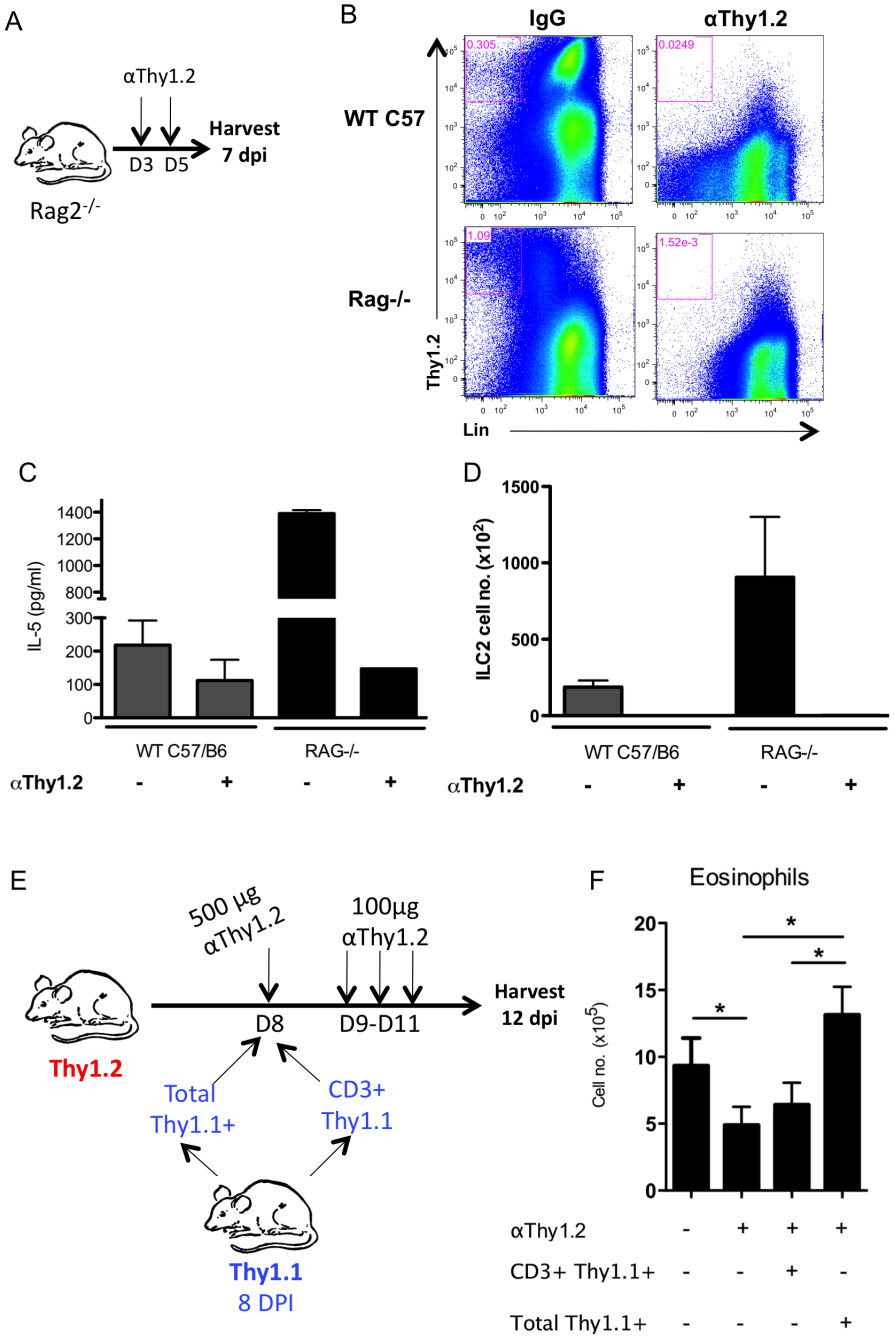
ILC2 are responsible for IL-5 production and eosinophil accumulation. (A–D) Rag2^−/−^ or C57BL/6 mice were infected with PR8 followed by i.p. injection of 500 µg of αThy1.2 depleting antibody at 3 and 5 d.p.i.. Lungs and BAL were analyzed at 7 d.p.i.. (B) Representative FACS plots of lung ILC2 and Thy1.2^+^ cell depletion. (C) BAL IL-5 and (D) absolute number of ILC2 following protocol described in (A). (E) Strategy of depleting ILC2 during the resolution phase via administration of αThy1.2 at 8 d.p.i., followed by i.v. transfer of 2×10^6^ total Thy1.1+ cells (inclusive of ILC2) or CD3^+^Thy1.1^+^ (excluding ILC2) from the lungs of 8 d.p.i. congenic Thy1.1^+^ mice. 100 µg of αThy1.2 was given 9–11 d.p.i. in order to continuously deplete endogenous ILC2. Lungs were harvested at 12 d.p.i. (F) Total eosinophil numbers in the lung following (E). Data is representative of 2–3 independent experiments with n≥4 per group.

To determine whether ILC2 can produce IL-5 following influenza infection *in vivo*, we first analyzed IL-5 production and the contribution of ILC2 as a source of IL-5 in adaptive immune cell deficient, IAV infected Rag2-/- mice since the majority of Thy1.2^+^ cells in these mice are ILC2 [10], [12], [24]. Infected Rag2-/- were depleted of ILC2 *in vivo* by administration of a depleting anti-Thy1.2 antibody at 3 and 5 d.p.i. (Figure 3A). IL-5 levels in the BAL fluid and ILC2 numbers in the IAV infected lungs were assessed at 7 d.p.i. in infected Rag2 -/- and wild type (WT) mice, in order to determine the extent of antibody mediated depletion of these cells. Depletion of Thy1.2^+^ cells in both Rag2-/- and WT was >99% (Figure 3B and 3D). As Figure 3C demonstrates infected, untreated, Rag2-/- mice produced dramatically higher levels of IL-5 in the BAL than their infected WT counterparts (Figure 3C). This finding is consistent with the higher absolute number of ILC2 accumulating in the lungs of infected Rag2 deficient mice (Figure 3D). Importantly, *in vivo* depletion of ILC2 in Rag2-/- (and WT) mice likewise resulted in a reduction of IL-5 in the BAL fluid to background levels (Figure 3C). These results support the view that ILC2 are fully capable of producing IL-5 during IAV infection, and are most likely the major *in vivo* producers of IL-5 in the respiratory tract of IAV infected mice.

To establish a direct connection between ILC2 and the IL-5 mediated accumulation of eosinophils in the lungs during the resolution phase of IAV infection, we devised an *in vivo* Thy1.2^+^ cell depletion and reconstitution strategy (Figure 3E). Specifically, Thy1.2^+^ mice were sub-lethally infected with IAV and at 8 d.p.i. the mice were depleted of Thy1.2^+^ cells by administration of a depleting antibody. Two hours following Thy1.2^+^ cell depletion the infected recipient mice received an inoculum of 2×10^6^ Thy1.1^+^ cells isolated from the lungs of 8 d.p.i. congenic Thy1.1+ mice consisting of either total Thy1.1^+^ cells (including ILC2) or CD3^+^Thy1.1^+^ lung cells (deficient in ILC2). To inhibit the regeneration/recruitment of endogenous Thy1.2^+^ ILC2 the mice received additional daily treatment with the depleting anti-Thy1.2 antibody for 3 days prior to quantification of lung infiltrating eosinophils at 12 d.p.i. (Figure 3E). As Figure 3F demonstrates, only recipients of total Thy1.1+ cells (i.e. containing ILC2) had restored eosinophil numbers in the infected lungs following depletion of endogenous ILC2. These data are consistent with the view that ILC2 continue to produce IL-5 during the recovery phase and thereby facilitate eosinophil accumulation.

### ILC2 are responsive to an *in vivo* source of IL-33

**Figure 4:**
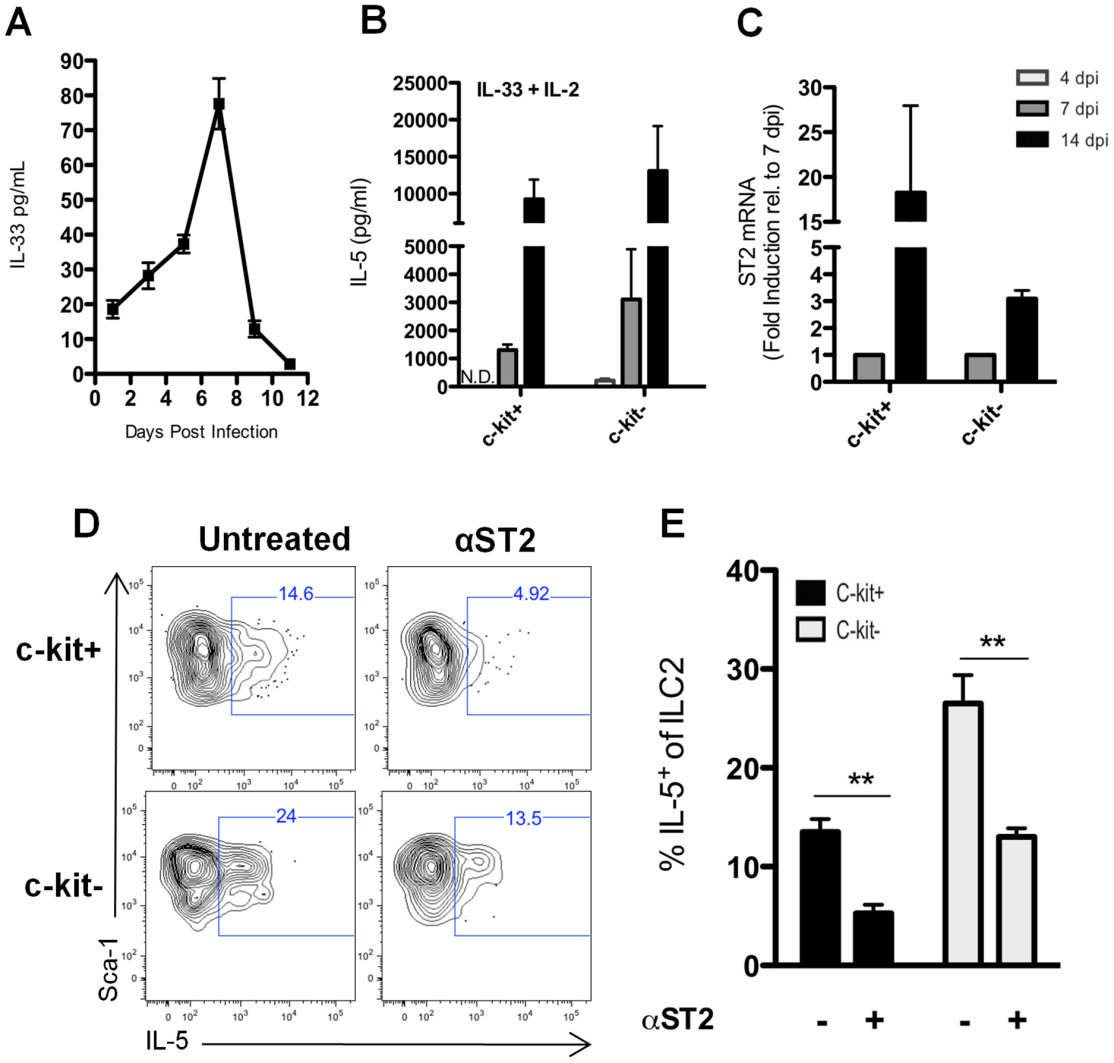
ILC2 are responsive to IL-33 present in the lung. (A) IL-33 present in the BAL was determined by Luminex at indicated d.p.i.. (B) c-kit^+^ and c-kit^−^ ILC2 were FACS sorted from the lung at the indicated d.p.i. and cultured in the presence of 10 ng/ml rIL-33 and 4 U rmIL-2 for 72 hr (ILC2 stimulated with IL-33 alone produced no detectable IL-5 protein – data not shown). Supernatants were collected and analyzed for IL-5 by ELISA. N.D. = Not detected. (C) c-kit^+^ and c-kit^−^ILC2 were FASC sorted from the lung at 7 and 14 d.p.i. (4 d.p.i. was not analyzed) and stored in trizol at −80 C. cDNA was made as described in Materials and Methods and ST2 gene expression was measured by RT-PCR. (D) Whole lung cell suspensions were cultured for 24 hours with or without 20 µg of ST2 blocking antibody. Golgi Stop was added for last fours hour of culture and intracellular IL-5 was assessed in ILC2 subsets. (E) Frequency of IL-5+ c-kit^+^ and c-kit^−^ ILC2 after blocking ST2 as in (D). Error bars are +/− SEM. n = 3–5 per group. (B–C) cells were FACS sorted from pooled lungs of 10–15 mice per day. Data are representative of 3 independent experiments. **p<.01.

ILC2 have been reported to produce IL-5 following exposure to several cytokines, most notably IL-33 and IL-25 [10], [25]. IL-33 is released from dead cells and infected cells including respiratory epithelial cells infected by IAV *in vivo* and *in vitro* [26]. Indeed, we could readily detect IL-33 (but not IL-25, Figure S6) in the BAL fluid from IAV infected lungs (Figure 4A). IL-33 was detectable as early as day 1 p.i., reached a maximum on day 7 p.i., and was still detectable in the BAL fluid of mice as late as day 11 p.i. (Figure 4A). In order to directly assess the impact of IL-33 on IL-5 production by ILC2, we isolated c-kit^+^ and c-kit^−^ ILC2 via FACS from IAV infected lungs at 4, 7, and 14 d.p.i. and stimulated them directly *ex vivo* with recombinant murine IL-33 (rIL-33) for 72 hours. Both c-kit^+^ and c-kit^−^ ILC2 isolated at 14 d.p.i. produced high levels of IL-5 (Figure 4B) in keeping with the evidence implicating IL-33 as an extremely potent stimulus for IL- 5 production by ILC2. IL- 5 production by ILC2 on a per cell basis was considerably lower (∼10 fold) for both c-kit^+^ and c-kit^−^ ILC2 isolated at 7 dp.i. and was either minimal (c-kit^−^ ILC2) or not detectable (c-kit^+^ ILC2) when cells were isolated and stimulated at 4 d.p.i. (Figure 4B). A likely explanation for the difference in sensitivity of ILC2 isolated from the infected lungs at different times p.i. to rIL-33 was suggested by our analysis of the level of expression of the gene encoding the ST2 subunit of the IL-33 receptor (IL-33R, Figure 4C). As with IL- 5 production, ST2 expression was undetectable on ILC2 isolated at 4 d.p.i., increased at 7 d.p.i. and was highest on ILC2 isolated at 14 (Figure 4C). These findings were consistent with the concept that IL-33 serves as a regulator of IL-5 expression by ILC2 during IAV infection and that the impact of IL-33 on IL-5 expression was likely to be most pronounced during the later phase of IAV infection.

In order to further establish the contribution of IL-33 as a stimulus for the production of IL-5 in the IAV infected lungs we developed an *ex vivo* intracellular cytokine staining (ICS) assay to detect and quantitate IL-5 producing ILC2. Total lung cell suspensions from IAV infected lungs were cultured for 24 hours in the presence or absence of blocking ST2 (IL-33R) antibody. IL-5 producing ILC2 present in infected lung cell suspensions were then enumerated by ICS (Figure 4D and 4E). As Figures 4D and 4E demonstrate, blockade of the IL-33 receptor ST2 subunit during *in vitro* culture of lung cell suspensions reduced the frequency of IL-5 producing ILC2 for both the c-kit^+^ and c-kit^−^ lung ILC2 subsets. This finding further implicates IL-33 produced *in vivo* during IAV infection as an important regulator of IL-5 production by ILC2.

### Sources of IL-33 in the IAV infected lungs

**Figure 5:**
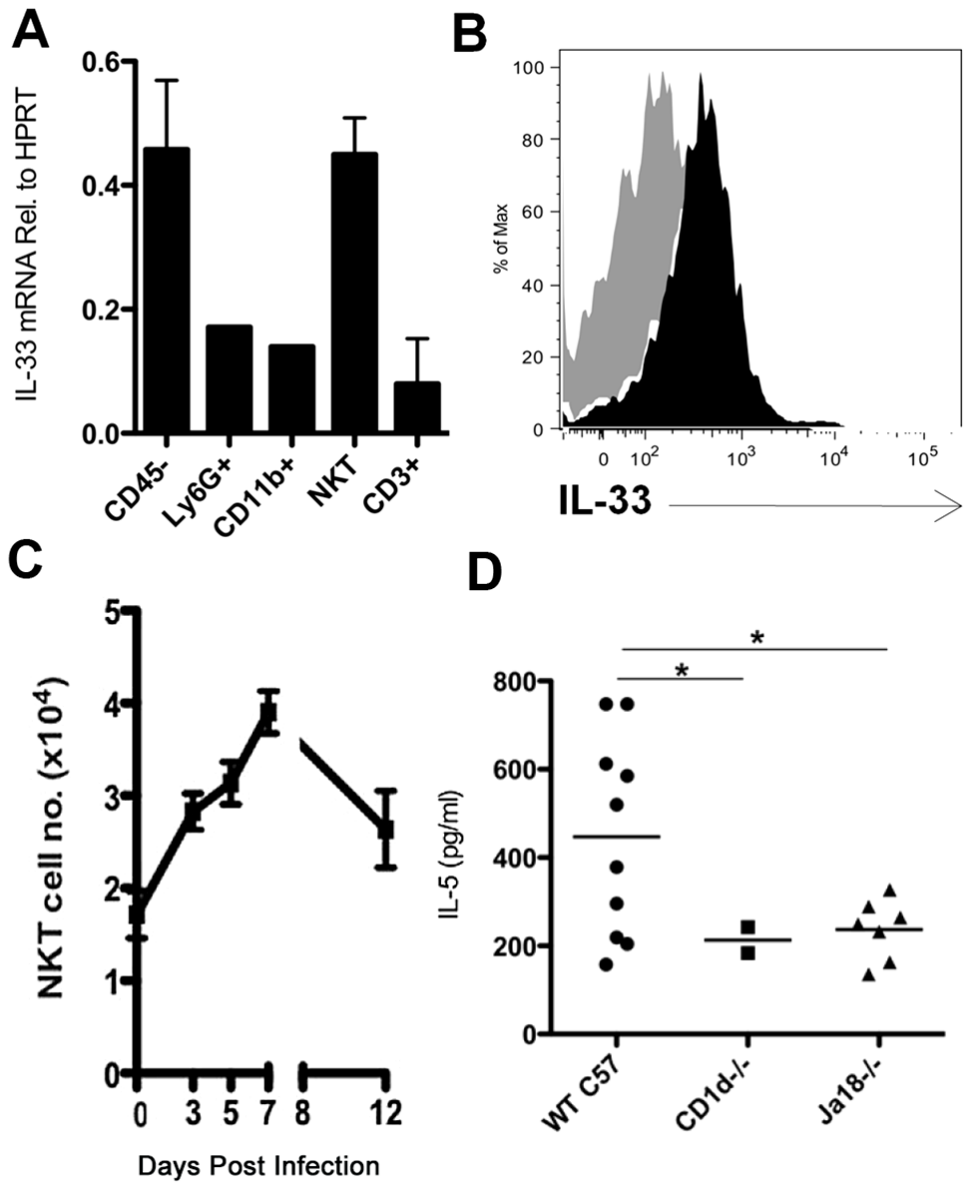
NKT are an *in vivo* source of IL-33. (A) IL-33 mRNA found in cell populations FACS sorted from the lung at 12 d.p.i.. (B) IL-33 protein in NKT as detected by intracellular staining (filled) compared to FMO control (shaded gray) at 12 d.p.i. (C) Total NKT cells identified in the influenza-infected lung as defined by CD3 and CD1d tetramer staining. (D) BAL IL-5 levels at 7 d.p.i. in indicated mouse strains. Data is representative of at least 2 independent experiments with 2–3 mice each. mRNA was collected from pooled, sorted cell populations from >5 mice. Bars = +/− SEM. *p<.05.

As noted above, IL-33 has been reported to be produced by a variety of cell types [27] and its expression in and release from injured/stressed cells –including epithelial cells–suggests the role of the cytokine as “alarm” signal released during cell death [28]–[30]. To identify potential sources of IL-33 in the IAV infected lungs we initially analyzed IL-33 gene expression in various cell types isolated from the infected lungs at 12 d.p.i. IL-33 gene expression was prominent both in the CD45^−^ fraction of whole lung cell suspensions (which includes infected respiratory epithelial cells undergoing virus induced as well as immune mediated cell death[18]), as well as, surprisingly, NKT cells (Figure 5A). Both CD45^−^ and NKT cells were also prominent sources of IL-33 at 7 d.p.i. (data not shown). NKT expressed and secreted IL-33 protein both at 7 and 12 d.p.i. (Figure S7 and Figure 5B). Although IL-33 has documented effects on NKT cells [31], [32], NKT had not heretofore been implicated as a source of IL-33, particularly during IAV infection. Lung infiltrating NKT cells increased in numbers over the course of infection reaching a maximum at 7 d.p.i. and only gradually decreased in numbers up to 12 d.p.i. (Figure 5C).

### NKT cells and the regulation of ILC2 IL-5 production

The above observations raised the possibility that NKT cells may regulate IL-5 production by ILC2. To explore this possibility, we first analyzed IL-5 release into the BAL fluid of control and invariant NKT cell deficient (Jα18-/-) and global NKT deficient (CD1d-/-) mice undergoing IAV infection. As Figure 5D demonstrates, IL-5 production was significantly decreased at 7 d.p.i. in Jα18-/- and CD1d-/- mice relative to infected WT mice.

**Figure 6:**
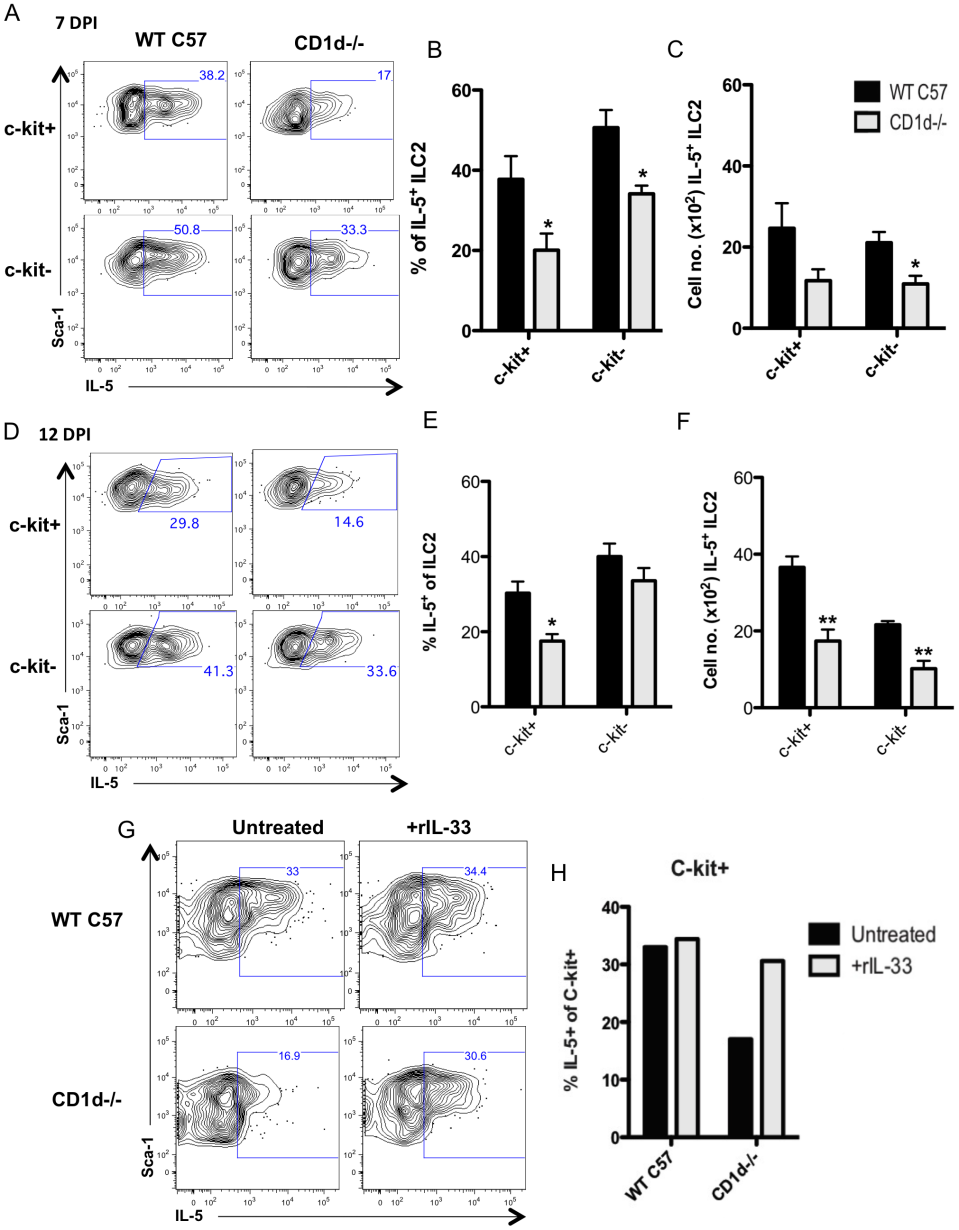
NKT cell deficient mice have fewer IL-5 producing ILC2. Lungs from infected CD1d^−/−^ or C57BL/6 (WT C57) were harvested at 7 (A–C) and 12 d.p.i. (D–E). (A) IL-5 production was measured in each ILC2 subset via the *ex vivo* intracellular cytokine assay (B) Total frequency and (C) number of IL-5+ c-kit^+^ or c-kit^−^ ILC2 at 7 d.p.i.. (D) ILC2 subsets from 12 d.p.i. lungs were examined as in (A). (E) Frequency and (F) total number of IL-5+ ILC2 subsets at 12 d.p.i.. (G) Whole lung cell suspensions from WT or CD1d^−/−^ mice at 12 d.p.i. were treated with 20 ng/ml rIL-33 for 24 hours in culture and analyzed for intracellular IL-5 in ILC2. (H) Percentage of IL-5+ c-kit^+^ ILC2 after treatment with rIL-33. Data is representative of 2 (G–H) and 4 (A–F) independent experiments with 2–4 mice per group. Bars = +/− SEM. *p<.05, **p<.01.

To more directly assess the impact of NKT cell deficiency on IL-5 production by ILC2 during infection, we examined ILC2 IL-5 production in total lung cell suspensions from WT and CD1d-/- mice at 7 and 12 d.p.i. using our *ex vivo* ICS assay. As Figures 6A and 6B demonstrate, the frequency of IL-5 producing ILC2 was significantly diminished at 7 d.p.i. in both c-kit^+^ and c-kit^−^ ILC2 from the infected NKT cell deficient donors. A corresponding decrease in the absolute number of IL-5+ ILC2 from lungs of infected CD1d-/- mice was likewise observed (Figure 6C). There was, similarly, a significant decrease in the frequency and number of IL-5 producing ILC2 among the more abundant c-kit^+^ ILC2 isolated from lungs of infected NKT cell deficient CD1d-/- mice at 12 d.p.i. (Figure 6D, 6E, and 6F). Although there was also a diminution in the frequency of IL-5+ c-kit^−^ ILC2, this did not achieve statistical significance (Figure 6E).

In view of these findings it was of interest to determine if addition of IL-33 to the lung cell cultures from NKT cell deficient donors could reverse the deficit in the frequency of IL-5 producing ILC2. As Figures 6G and 6H demonstrate, addition of rIL-33 to 12 d.p.i. lung cell suspensions at the start of the 24-hour *ex vivo* culture system resulted in an increase in the frequency of IL-5+ ILC2 among the more abundant c-kit^+^ ILC2 to levels comparable to that of NKT cell sufficient WT mice, indicating that IL-33 can compensate for the decrease in the frequency of IL-5+ ILC2 resulting from NKT cell deficiency. Of note, IL-33 supplementation had no effect on the frequency of IL-5+ ILC2 in lung cell suspensions from control WT mice, suggesting that endogenous IL-33 may be at a saturating level in WT lung suspensions.

**Figure 7:**
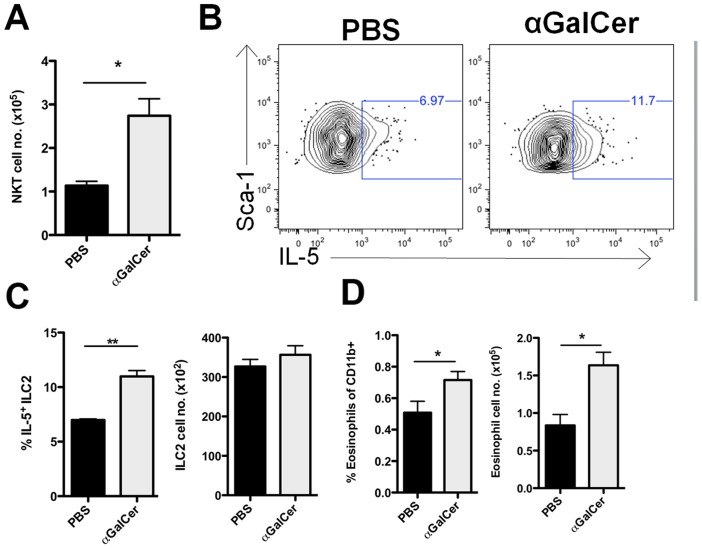
αGC administration increases both IL-5+ ILC2 and eosinophil numbers in the lungs. (A) 1 µg/ml αGC was injected i.p. at 3 and 5 d.p.i.. Lungs were harvested at 7 d.p.i. and total NKT cells were quantified by use of CD3 and CD1d tetramer staining. (B) Representative FACS plots of intracellular IL-5 after 24 hours of *ex vivo* culture. (C) Frequency of IL-5+ ILC2 (left panel) and absolute number of ILC2 at 7 d.p.i. (right panel). (D) Frequency (left panel) and absolute number (right panel) of eosinophils detected in the lung at 7 d.p.i.. Data is representative of 2 independent experiments with 3 mice each. Bars = +/− SEM. *p<.05, **p<.01.

Alpha-galactosylceramide (αGC) is an extremely potent and specific activator of NKT cells [33]. If NKT cells are an important contributor to the control of IL-5 production by ILC2 then augmenting NKT cell numbers and function by αGC administration would be expected to enhance ILC2-dependent IL-5 production during IAV infection. αGC administration to IAV infected WT mice at 3 and 5 d.p.i., resulted in a 2–3 fold increase of NKT cells in the infected lungs at 7 d.p.i. (Figure 7A). Consistent with the above findings, we observed an increase in the frequency of IL-5 producing ILC2 in 7 d.p.i. *ex vivo* lung cultures from αGC treated infected WT mice (Figure 7B and 7C) but no increase in the absolute number of ILC2. The increase in IL-5 producing ILC2 following αGC administration coincided with an increase in both the frequency and absolute numbers of eosinophils in the treated IAV infected lungs (Figure 7D).

### NKT and alveolar macrophages support ILC2-derived IL-5 production

**Figure 8:**
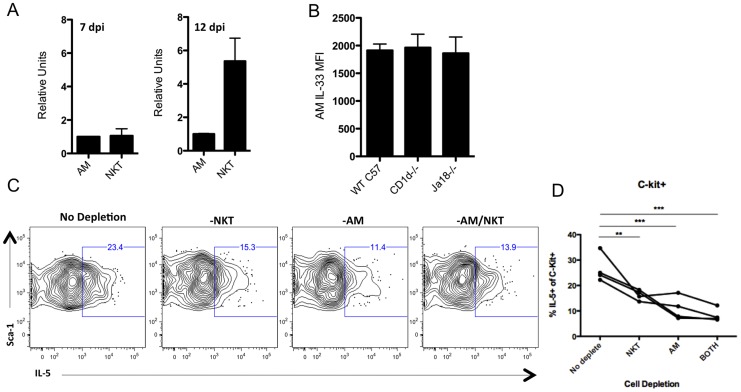
NKT cells regulate ILC2 derived IL-5 production independently of alveolar macrophages. (A) Alveolar macrophages (AM) and NKT were FACS sorted from the lungs at the indicated days and analyzed for IL-33 mRNA via RT-PCR. Cells were pooled from >5 mice for each group at each day. (B) AM were analyzed for intracellular IL-33 protein at 12 d.p.i. in indicated mouse strains. (C) Representative FACS plots of intracellular IL-5 in c-kit^+^ ILC2 at 12 d.p.i. after 24 hour *ex vivo* culture with or without indicated cell depletion. (D) Frequency of IL-5+ c-kit^+^ ILC2 at 12 d.p.i. following *ex vivo* cell depletion after 24 hour culture. Data is representative of 3 experiments with 4–10 mice each. Bars = +/− SEM. **p<.01, ***p<.001.

Alveolar macrophages (AM) were recently shown to be a likely source of the IL-33 that regulates IL-13 production by ILC2 during IAV [13]. IL-33 gene expression is induced in both NKT and AM following IAV infection (Figure S8). AM isolated at 7 d.p.i. indeed expressed IL-33 transcripts at levels comparable to that of NKT cells, however, AM isolated from 12 d.p.i. lungs expressed approximately one fifth the level of IL-33 mRNA as NKT cells (Figure 8A). It was recently noted that NKT cells can influence the level of IL-33 in AM [34], however, the expression level (MFI) of IL-33 protein in 12 d.p.i. AM from WT and NKT cell deficient (CD1d-/- or Jα18) mice was comparable (Figure 8B) suggesting that the diminished IL-5 response in NKT cell deficient mice was not linked to decreased IL-33 expression by AM.

To further assess the contribution of NKT cells and AM in the production of IL-5 by c-kit^+^ ILC2 in the IAV infected lungs we analyzed the impact of selective depletion of NKT cells and/or AM from 12 d.p.i. total lung cell suspensions prior to 24-hour *ex vivo* culture on the frequency of IL-5^+^ c-kit^+^ ILC2 (the predominant ILC2 type at this time after infection). Depletion of either cell type from the lung cell suspension resulted in ∼40%–50% reduction in IL-5 producing ILC2 (Figure 8C and 8D). Of note, simultaneous depletion of both cell types did not result in a further significant reduction in the frequency of IL-5^+^ ILC2, suggesting that other factors may also regulate IL-5 production by ILC2.

## Discussion

IL-5 has been previously reported to be produced in the lungs during experimental IAV infection [15]–[17]. In the current study we focused on defining the cellular source of IL-5 in the influenza-infected lungs and the tempo of IL-5 protein and gene expression. We observed that the peak of IL-5 release into the airways (as detected in BAL fluid) corresponded to the peak of effector T cell dependent pro-inflammatory cytokine release into the BAL i.e. 6–8 d.p.i., while IL-5 gene expression persisted for a longer time frame i.e. 11–13 d.p.i. IL-5 production was associated with the progressive increase in eosinophils infiltrating the infected lungs particularly during the recovery phase, that is following infectious virus clearance (8–10 d.p.i.; [18]).We went on to demonstrate that the source of this IL-5 produced in the lungs during IAV infection is primarily the recently described group 2 innate lymphoid cell (ILC2) [9]. We found that both c-kit^+^ and c-kit^−^ ILC2 accumulate in the lung throughout the course of infection, however, these two ILC2 subsets appear to be recruited in a biphasic manner, that is c-kit^−^ ILC2 are predominant during the acute phase of infection (0–7 d.p.i.) while the c-kit^+^ dramatically increase during the recovery phase (Figure 2B). As has been previously reported [10], ILC2 IL-5 production is regulated by IL-33, one source of which we now identify as the NKT cell. In support of this conclusion we found that NKT cell deficient mice have less IL-5 present in the BAL at 7 d.p.i. as well as significantly diminished IL-5 production from ILC2 during the recovery phase at 12 d.p.i.. Conversely, treatment of wild type mice with the NKT cell activating ligand αGC significantly increased IL-5 production from ILC2 and subsequently increased eosinophil numbers by 2-fold.

Our finding that ILC2 are the major source of the type 2 cytokine IL-5 (as well as IL-13, data not shown) and that ILC2 produce extremely high levels of this cytokine on a per cell basis, are in keeping with recent findings on type 2 cytokine production by ILC2 in a model of experimental helminth infection [10], [12]. Similarly, ILC2 were implicated as the primary source of the type 2 cytokine IL-13 in a model of experimental IAV infection [13], as well as major producers of the epidermal growth factor family member, amphiregulin [14]. We detected elevated levels of amphiregulin in ILC2 during the recovery phase, consistent with the role of ILC2 in maintaining epithelial barrier integrity (Figure S4). In the current study, we also analyzed the tempo of accumulation of c-kit^+^ and c-kit^−^ ILC2 in the IAV infected lungs. As noted above, c-kit^+^ ILC2 are the predominant IL-5 producing cell type during the late, “recovery” phase of infection, that is following virus clearance. Although there is abundant expression of the c-kit ligand, stem cell factor, in the IAV infected lungs (data not shown) we could not demonstrate by several criteria (Ki67 expression and BrdU uptake) local proliferation of the c-kit^+^ ILC2 in the infected respiratory tract. Therefore, the marked accumulation of this ILC2 subset in the infected lungs during the recovery phase of infection most likely reflects the recruitment of the cell type from bone marrow stores [24], [35]. At present, it is not clear why the tempo of accumulation of c-kit+ and c-kit^−^ ILC2 differs during the course of infection as we detect no difference in the intrinsic capacity of these two ILC2 subsets to produce IL-5. Since current evidence suggests that this novel lymphoid like cell type arises from progenitors in the bone marrow [24], [35], differences in the type or magnitude of cytokines produced within the IAV infected lungs at different points in the course of infection may regulate the preferential recruitment and accumulation of one or the other ILC2 subset.

IL-33 (along with IL-25) has been reported to be a regulator of type 2 cytokine production by ILC2 [10], [12], [25]. Indeed in a recent analysis of the role of IL-33 in the regulation of type 2 cytokine production by ILC2, alveolar macrophages (AM) were implicated as the source of IL-33 regulating ILC2 cytokine production [13]. In the current report we demonstrate that NKT cells infiltrating the IAV infected lungs are another source of IL-33 influencing the magnitude of type 2 cytokine, in this case IL-5, production. While NKT cells and AM expressed comparable levels of IL-33 mRNA at 7 d.p.i., IL-33 transcript levels were up to four-fold higher in NKT cells at 12 d.p.i. However, analysis of the contribution of each of these innate immune cell subsets in the regulation of IL-5 production by ILC2 in our *in vitro* lung cell suspension culture system suggests that, at 12 d.p.i. at least, NKT cells and AM may have a similar impact on ILC2 cytokine production. It is noteworthy that simultaneous depletion of both innate immune cell subsets from lung cell suspensions did not have an additive effect on the frequency of IL-5+ILC2. Since multiple cell types, including as noted in this report CD45^−^ cells, from infected lungs can produce IL-33 [27], it is likely that IL-33 produced by one or more other cell types can in part compensate for the loss of IL-33 resulting from the elimination of NKT cells (and/or AM) *in vitro*. Thus, our data favors the view that multiple cell types within the IAV infected lungs act in concert to regulate type 2 cytokine production by ILC2 through active secretion of IL-33.

Pulmonary eosinophilia is not usually considered to be a feature of the strong type 1 immune response elicited by infection with potent respiratory viral pathogens like IAV. Nevertheless, in this report we (and others [36]) have documented the influx of eosinophils into the lungs during experimental IAV infection as well as peripheral eosinophilia under certain circumstances in human IAV infection [37]. Our analysis of the impact of IL-5 neutralization *in vivo* on eosinophil accumulation in the infected lungs implicates pulmonary IL-5 as a critical controller of eosinophil influx during IAV infection. Our IL-5 neutralization experiments also suggest a critical role for the cytokine in orchestrating the progressive accumulation of eosinophils in the lungs following virus clearance when IL-5 is no longer detectable in BAL fluid. It is also likely that other mediators (or lack thereof), such as eotaxins (along with the precipitous drop in the production of pro-inflammatory, eosinophilia suppressing cytokines such as IFNγ following IAV clearance in the lungs), work in concert with IL-5 to enhance eosinophil accumulation in the lung during the recovery phase [38].

Several factors likely account for our inability to detect IL-5 in the airways at later time points during the resolution phase of infection. Following virus clearance there is regeneration/restoration of the airway basement membrane and respiratory epithelial cell barrier integrity. This would certainly limit the extent to which soluble mediators like IL-5 could “leak” into the airways. A more likely possibility is that IL-5 produced by ILC2 is taken up and consumed by IL-5 receptor expressing cells, most notably eosinophils. Whether other cell types express a functional IL-5 receptor in the IAV infected lungs and therefore also contribute to the consumption of this cytokine during the resolution phase of infection remains to be determined.

In the setting of underlying reactive airway disease, respiratory virus infections are strongly linked to subsequent asthma exacerbation [39], [40]. The proinflammatory cytokines produced in response to respiratory viral infection can enhance airway hyperreactivity and damage the sensitized respiratory epithelial barrier. Type 2 cytokines e.g. IL-4, IL-13, if produced during respiratory viral infection, are capable of promoting airway smooth muscle contraction and increased mucus secretion. Indeed, Chang et al. in their recent analysis of IL-13 production by ILC2 during IAV infection have suggested that this ILC2 derived IL-13 triggers airway hyperreactivity and therefore may represent an important mechanism resulting in asthma exacerbation during IAV infections. They did not, however, find any eosinophil accumulation in their model of influenza infection (using the reassortant Mem/71/Bel strain of IAV). On the other hand, in this report we demonstrate that ILC2 derived IL-5 is critical for eosinophil accumulation during IAV infection with A/PR8/34. The reason for the difference in eosinophil accumulation in these two reports is uncertain but differences in the properties of the two IAV strains employed for infection likely contributes.

Allergic airway disease is characterized by respiratory tract eosinophilia and eosinophils are proposed to play a role in asthma exacerbation by stimulating mucus hyper secretion and enhanced airway hyperreactivity [3]. At present, the precise role of eosinophils in the host response to IAV infection is uncertain. Thus this cell type may contribute to enhanced airway disease as proposed for IL-13 [13]. On the other hand eosinophils have been reported to have an antiviral role through release of secreted products [41], [42]. Such a role for the cell type in experimental IAV infection seems unlikely since maximum accumulation of eosinophils in the pulmonary interstitium occurs after infectious virus clearance. On the other hand, the tempo of eosinophil accumulation and increased IL-5 production during the resolution phase of infection raises the possibility that this type 2 response could have a positive role in restoration of pulmonary architecture and function by promoting tissue repair [43].

We have analyzed recovery from IAV infection in eosinophil deficient PHIL mice [3] and observed no effect of eosinophil deficiency on susceptibility to lethal infection or recovery from infection as monitored by restoration of weight and parameters of pulmonary inflammation (Gorski, Hahn and Braciale, unpublished observations). These results, while arguing against a critical role for eosinophils in the initial stages of restoring pulmonary structure and function following IAV clearance, do not exclude the possibility of a more subtle role for eosinophils at late times in the process of recovery from the IAV infection. Indeed, we observed a decrease, although not significant, in pulmonary lung function following IL-5 neutralization. Furthermore, IL-5 has been demonstrated to serve a protective role, independent of eosinophils, in experimental polymicrobial sepsis [44], suggesting that IL-5 may act on other lung cell targets to promote restoration of pulmonary structure and function following IAV infection. Additional studies will need to be undertaken to better understand the contribution of eosinophils and/or IL-5 to the host response to IAV infection.

In conclusion, we have demonstrated that ILC2 are the cellular source of IL-5 produced during experimental IAV infection. The production of IL-5 by ILC2 is essential for the gradual, progressive infiltration and accumulation of eosinophils into the infected lungs. We demonstrate that one of the important sources of IL-33, an essential regulator of IL-5 production by ILC2, is the NKT cell. The precise role of eosinophils and IL-5 in the host response to IV infection is currently being evaluated.

## Materials and Methods

### Ethics statement

All animal experiments conducted in this study were carried out in accordance with the Animal Welfare Act (Public Law 91-579) and the recommendations in the Guide for the Care and Use of Laboratory Animals of the National Institutes of Health (OLAW/NIH, 2002). All experiments were approved by the University of Virginia Animal Care and Use Committee (Protocol Number 2230).

### Mice and infection

BALB/c and C57BL/6 mice were purchased from the National Cancer Institute and maintained at the University of Virginia in a pathogen-free environment. Mice deficient in CD1d and Rag2 were a kind gift from Drs. Mark Okusa and Timothy Bender (University of Virginia), respectively. Jα18^−/−^ mice were provided by Dr. Victor Laubach (University of Virginia), and TCRγδ-/- mice were purchased from Jackson Laboratories. All mice used in experiments were between the ages of 8–12 weeks and matched for age and sex. Type A influenza virus A/PR/8/34 (H1N1) was grown in day 10 chicken embryo allantoic cavities as described previously [45]. Mice were infected with 300 egg infectious doses (EID_50_) of A/PR/8/34 i.n. (corresponding to a 0.1 LD_50_ dose) unless otherwise stated.

### Preparation of lung tissue

Lungs were perfused with 5 mL of PBS via the right ventricle of the heart to remove cells from the vasculature. To prepare a single cell suspension, lungs were minced and digested in media containing 183 U/ml type II collagenase (Worthington) for 45 minutes at 37°C. Lung tissues were then pushed through a steel screen and red blood cells were lysed with ammonium chloride buffer (e.g. ACK lysis buffer). Cells counts were performed with a hemocytometer and trypan blue was used to exclude dead cells. To prepare total lung RNA, lungs were homogenized in 1 ml TRIzol (Invitrogen) immediately following perfusion and stored at −80°C until analyzed.

### Cytokine analysis of BAL fluid

Bronchoalveolar lavage (BAL) fluids were obtained by cannulating the trachea and flushing the lungs with 0.5 ml of sterile PBS three times. Cells were removed by centrifugation and supernatants were stored at −80°C until analyzed. IL-5 levels were quantified by either ELISA (eBioscience) or a multiplex Luminex assay (Flow Cytometry Core Facility at the University of Virginia). Cytokines such as IL-33, IL-13, and IL-25 were analyzed at various d.p.i. using the Luminex assay.

### RT-qPCR analysis

Total RNA was isolated from whole lung samples or FACS sorted cell populations frozen in TRIzol according to the manufacturer's instructions. RNA samples were treated with DNase I (Invitrogen) and complimentary DNAs (cDNA) were synthesized using random primers and Superscript II (all Invitrogen). Real time PCR of cDNA was performed in a StepOnePlus PCR system (Applied Biosystems) using SYBR Green master mix (Applied Biosystems). Primer sequences used were as follows: IL-5 F: 5′-GCTTCTGCACTTGAGTGTTCTG-3′, R: 5′-CCTCATCGTCTCATTGCTTGTC-3′ [46]; IL-33 primers F: 5′- TCCTTGCTTGGCAGTATCCA-3′, R: 5′-TGCTCAATGTGTCAACAGACG-3′; ST2 F: 5′-GCAATTCTGACACTTCCCATG-3′, R: 5′- ACGATTTACTGCCCTCCGTA-3′ [47]; Areg F: 5′-CTATCTTTGTCTCTGCCATCA-3′, R: 5′- AGCCTCCTTCTTTCTTCTGTT-3′ [48].

### In vivo antibody administration

Mice were given 100 µg of anti-IL-5 (TRFK5, eBioscience) or control IgG (Jackson ImmunoResearch) i.p. in 100 µl of sterile PBS at indicated days post infection. Mice were given 300 µg of anti-CD8α (2.43, BioExpress), 250 µg of anti-CD4 (GK1.5, BioExpress), or control IgG i.p. at 3 d.p.i.. In some experiments, 250 µg of anti-NK1.1 (PK136, a kind gift from Dr. Young Hahn, University of Virginia) or control IgG was given at 5 d.p.i.. Levels of IL-5 in the BAL were assessed at 7 d.p.i.

### Flow cytometry and antibodies

Single cells were suspended in FACS buffer containing PBS, 2% FBS, 10 mM EDTA, and 0.01% NaN_3_. Cell suspensions were blocked with anti–mouse CD16/32, followed by incubation with either specific mAbs or isotype-matched control Igs for 20 min at 4°C. The following antibodies were used: CD11c, CD11b, CD45, Sca-1, C-kit, CD44 (Biolegend); Ly6G, SiglecF, CD49b, CD3, CD4, CD8, CD19, TCRβ, TCRγ, NK1.1 (BD Biosciences); and Thy1.2 (eBioscience). Lineage cocktail included: CD3, CD4, CD5, CD8, CD11b, Gr-1, CD19, B220, DX5 (or NK1.1) and TCRδ. Proliferation was assessed by Ki-67 and BrdU staining (BD Biosciences). For BrdU staining, mice were injected with 500 µl of 1 mg/ml of BrdU i.p. Six hours later, lungs were harvested and cells were strained for intranuclear BrdU incorporation. Mice that were not injected with BrdU were used for a staining control. Production of IL-33 (R&D Systems) and IL-5 (Biolegend) were detected by intracellular cytokine staining using Cytofix/Cytoperm (BD Biosciences) as per the manufacturer's instructions. CD1d-tetramer was provided by the NIH Tetramer Core Facility. Flow cytometry was performed on FACSCanto II flow cytometer (BD Biosciences), and data were analyzed using FlowJo (Tree Star, Inc.).

### ILC2 cell culture *in vivo*

ILC2 were FACS sorted from lung suspensions using an iCyt Reflection sorter and kept in culture for 3 days in the presence of indicated stimuli in complete RPMI media supplemented with 10% FCS. 30 ng/ml of PMA and 500 ng/ml of ionomycin (both from Sigma) were used to stimulate ILC2. rIL-33 (Biolegend) and rIL-2 (eBioscience) were used at 10 ng/ml and 100 U/ml, respectively.

### *Ex vivo* intracellular cytokine assay

Single cell suspensions from whole lung were cultured for 24 hours at 37°C (approx. 5×10^6^ cells/ml) in culture medium containing 10% FCS. GolgiStop (1 µl/ml, BD Biosciences) was added for the last 4 hours of culture. Cells were surface stained for 15 minutes at room temperature, followed by fixation and permeabilization with Cytofix/Cytoperm (BD Biosciences). Intracellular cytokine staining for IL-5 was performed according the manufacturer's protocol. Anti-ST2 blocking antibody (Clone DJ8; MD Bioscience) was added to the culture wells at a concentration of 20 µg/ml.

### Thy1.2^+^ cell depletion *in vivo*

Rag2^−/−^ mice were given 500 µg of anti-Thy1.2 depleting antibody (30H12, BioXcell) at 3 and 5 d.p.i. i.p. For ILC2 depletion during the recovery phase, wild type C57BL/6 mice were given 500 µg of 30H12 antibody at 8 d.p.i., followed by daily i.p. injections of 100 µg (or equivalent amounts of control IgG).

### Pulse oximetry

BALB/c mice that had been treated with αIL-5 or IgG were anesthetized with bupivacaine. Blood oxygen saturation levels were measured using Mouse Ox Plus (Life Star Technologies). The oximeter clip was placed on the right thigh and blood oxygen saturation (SpO_2_) was recorded every .3 seconds. Overall SpO_2_ was determined by taking the average of all measurements once values reached a plateau and immediately prior to mice recovering from anesthesia.

### *In vivo* α-galactosylceramide (αGC) treatment

BALB/c mice were given 2 µg of αGC (a kind gift from Dr. Tsuji, NYU) or a control volume of PBS i.p. at 3 and 5 d.p.i.. Lungs and BAL were harvested at 7 d.p.i. and assessed for IL-5^+^ ILC2 (via *ex vivo* intracellular cytokine assay described above) and eosinophil infiltration into the lung.

### *Ex vivo* depletion of alveolar macrophages and NKT

For *ex vivo* removal of NKT cells and alveolar macrophages (AM) from whole lung cell suspensions, cells were incubated with APC-conjugated CD1d-Tetramer (NIH tetramer facility) or PE conjugated anti-SiglecF antibody, respectively. Cells were incubated for 15 min at 4°C then washed with an excess amount of FACS buffer (PBS+10% PBS+10 mM EDTA). Cells were then incubated with anti-APC or anti-PE magnetic beads (Miltenyi Biotec), kept at 4°C for 20 minutes, followed by washing in FACS buffer. NKT cells and AM were then removed from the suspensions by putting cells through a MACS Separation LS Colum (Miltenyi Biotec). Depletion efficiency was confirmed via FACS analyses of the collected samples. The remaining cells were resuspended in culture media for direct *ex vivo* intracellular cytokine analysis as described above.

### Statistical analysis

Statistical analyses were performed using Prism 5 (GraphPad Software). Unpaired, two-tailed Student t test or one-way ANOVA with a Tukey post test were used to determine significance. P values<.05 were considered statistically significant.

### Gene ID

The following are NCBI gene IDs for the genes analyzed: IL5: 16191, IL-33: 77125, IL-25: 140806, amphiregulin: 11839.

## Supporting Information

Figure S1 **Eosinophil numbers rebound following early cessation of αIL-5 treatment.** (A) C57BL/6 mice were given 100 µg of neutralizing anti-IL-5 antibody (αIL-5) i.p. daily from 7–10 d.p.i. with lungs being analyzed at 14 d.p.i.. (B) Representative flow plots of 14 d.p.i. lungs of mice given control IgG or αIL-5 as described in (A). Eosinophils were identified as CD45^+^SiglecF^+^CD11c^lo^.
(TIF)

Figure S2 **IL-5 transcripts are present in a Thy1.2^+^ non-canonical cell population.** (A) Indicated cell populations were sorted from the lungs of 5 d.p.i. mice and analyzed for IL-5 transcript via RT-PCR. (B) Lymphocytes thought to make up the Thy1.2^+^ population were FACS sorted from 7 d.p.i. lung. ND = Not detected. (C) C57BL/6 mice were given indicated depleting antibodies as described in Materials and Methods and BAL was collected at 7 d.p.i. for measurement of IL-5 protein by ELISA. (D) BAL from indicated knockout mouse strains was collected at 7 d.p.i. and analyzed for IL-5 protein. (A–B) from pooled mice, n = 5, (C–D) n = 3–5 per group.
(TIF)

Figure S3 **Surface marker expression of ILC2 subsets.** Lung c-kit^+^ (black line) and c-kit^−^ (blue line) ILC2 subsets were analyzed for indicated surface markers between 10–12 d.p.i.. Isotype controls are represented as shaded histrograms.
(TIF)

Figure S4 **ILC2 express amphiregulin.** ILC2 subsets were FACS sorted from the lung and analyzed for amphiregulin (areg) transcripts at indicated d.p.i.. N.D. = not determined.
(TIF)

Figure S5 **Group 2 innate lymphoid cells do not proliferate in the respiratory tract.** (A) ILC2 subsets from 10 d.p.i. lung were intracellularly stained for the proliferation marker Ki67. (B) 7 d.p.i. mice were injected with BrdU 4 hours before harvesting the lungs and staining for BrdU.
(TIF)

Figure S6 **IL-25 is not detectable in the BAL during IAV infection.** C57BL/6 mice were infected with PR8 and BAL fluid harvested at the indicated d.p.i.. Protein analyzed via Luminex. Limit of detection = .08 pg/ml.
(TIF)

Figure S7 **NKT cells secrete IL-33 protein.** (A) NKT cells were MACS enriched from 7 d.p.i. lung cell suspensions (purity >92%) and cultured (2×10^5^ cells/well) with or without BMDC and/or 10 ng/ml αGalCer for 24–48 hours. Supernatants were analyzed for IL-33 via ELISA (Biolegend). (B) Intracellular IL-33 was analyzed in NKT cells from 12 d.p.i. lung cell suspensions cultured for 24 hours *ex vivo* with or without GolgiSTOP added for the last 4 hours of culture. n = 5–6 per group. Bars = +/− SEM. BMDC = bone marrow dendritic cell, n.s. non-significant. **p<.01, ***p<.001 (compared to BMDC alone).
(TIF)

Figure S8 **IAV infection induces IL-33 expression in alveolar macrophages and NKT cells.** Alveolar macrophages (AM) and NKT cells were FACS sorted from the lung at indicated d.p.i. and analyzed for IL-33 transcript levels. Cell from n = 5–15 pooled lungs per day.
(TIF)
