# Supplementary figures and images for: Group 2 Innate Lymphoid Cell Production of IL-5 Is Regulated by NKT Cells during Influenza Virus Infection
Source: PLoS Pathog. 2013 Sep 19;9(9):e1003615. doi: 10.1371/journal.ppat.1003615 (PMC3777868; doi:10.1371/journal.ppat.1003615)

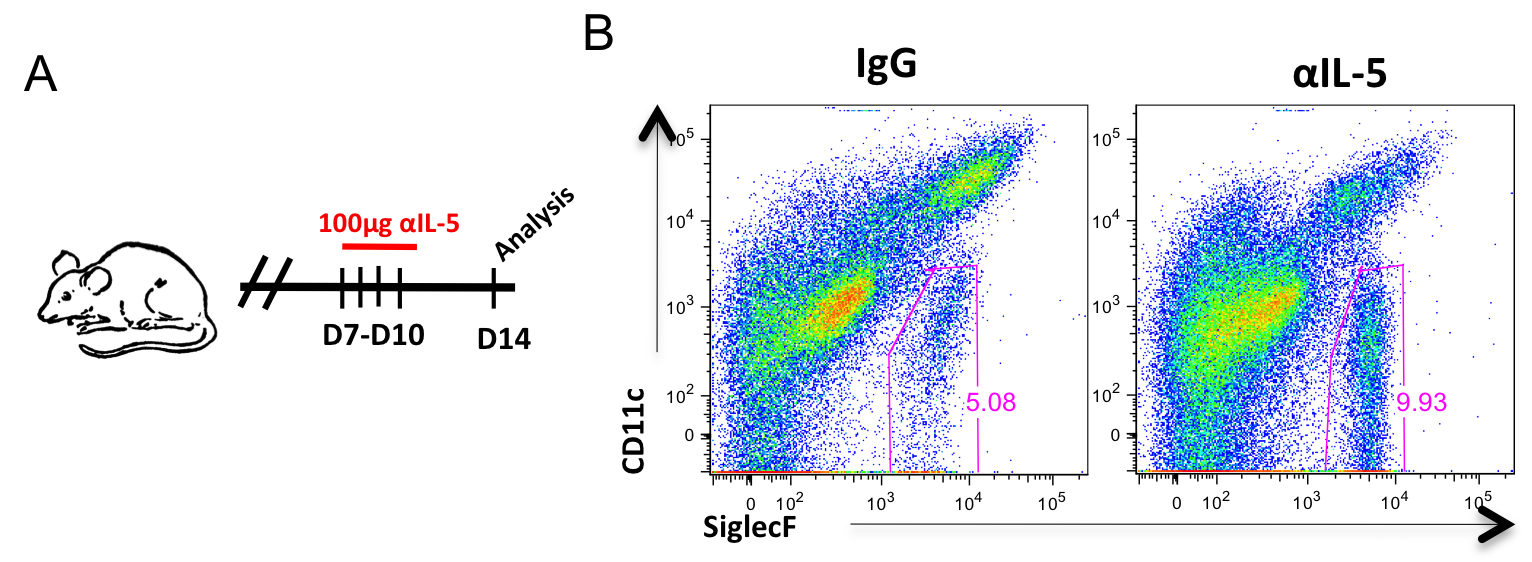

Supplement: Figure S1 — Eosinophil numbers rebound following early cessation of αIL-5 treatment. (A) C57BL/6 mice were given 100 µg of neutralizing anti-IL-5 antibody (αIL-5) i.p. daily from 7–10 d.p.i. with lungs being analyzed at 14 d.p.i.. (B) Representative flow plots of 14 d.p.i. lungs of mice given control IgG or αIL-5 as described in (A). Eosinophils were identified as CD45+SiglecF+CD11clo. (TIF) [file ppat.1003615.s001.tif]

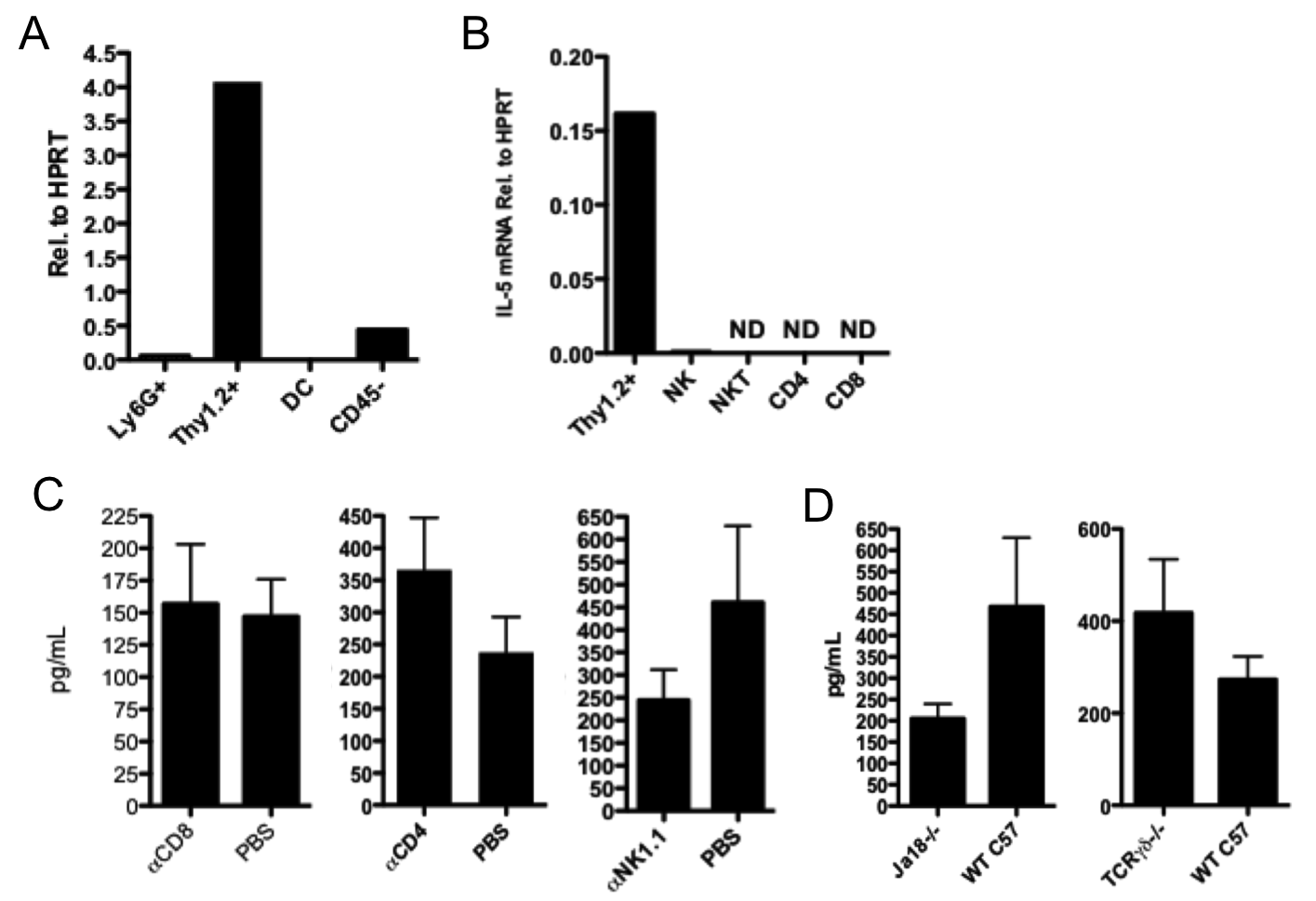

Supplement: Figure S2 — IL-5 transcripts are present in a Thy1.2+ non-canonical cell population. (A) Indicated cell populations were sorted from the lungs of 5 d.p.i. mice and analyzed for IL-5 transcript via RT-PCR. (B) Lymphocytes thought to make up the Thy1.2+ population were FACS sorted from 7 d.p.i. lung. ND = Not detected. (C) C57BL/6 mice were given indicated depleting antibodies as described in Materials and Methods and BAL was collected at 7 d.p.i. for measurement of IL-5 protein by ELISA. (D) BAL from indicated knockout mouse strains was collected at 7 d.p.i. and analyzed for IL-5 protein. (A–B) from pooled mice, n = 5, (C–D) n = 3–5 per group. (TIF) [file ppat.1003615.s002.tif]

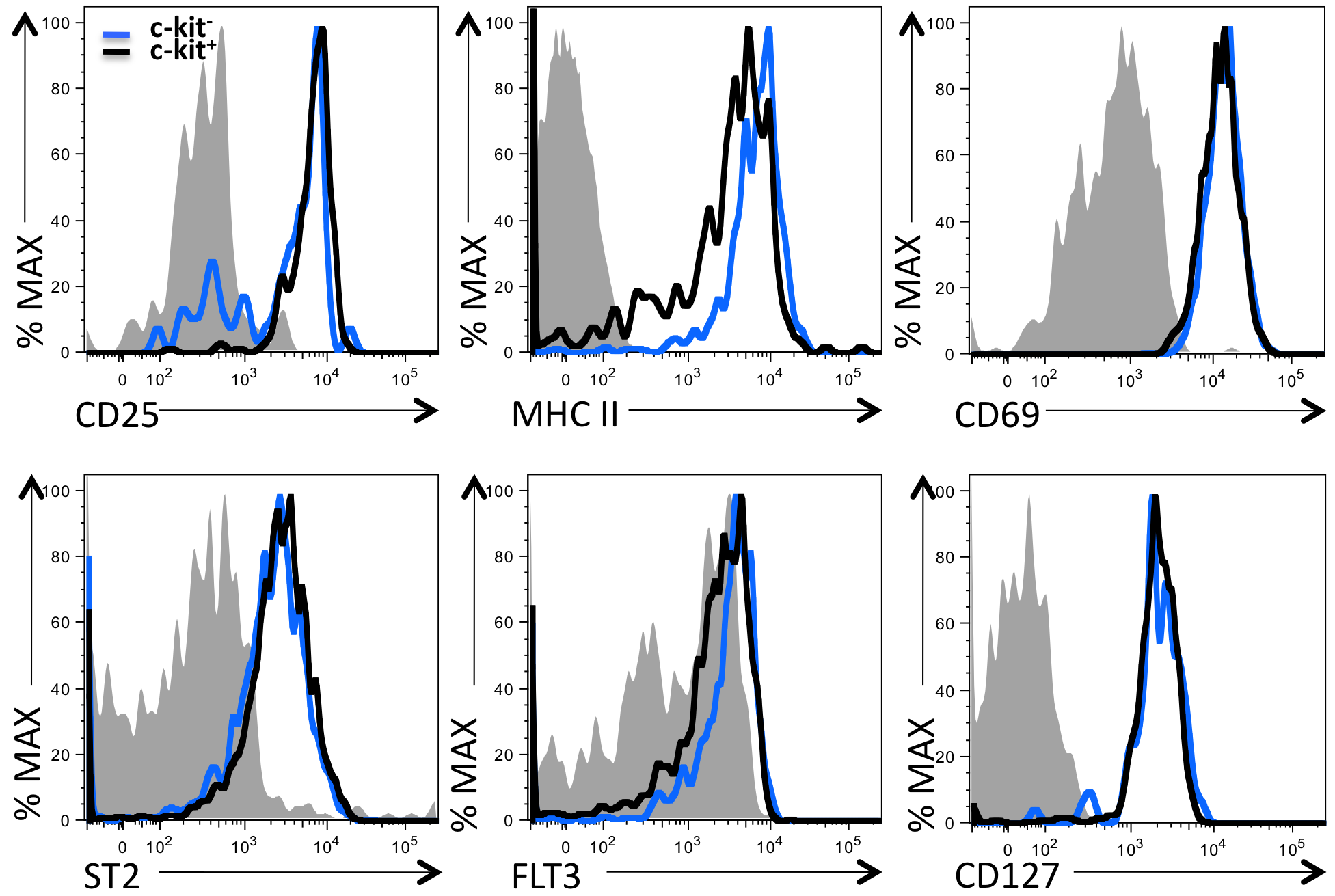

Supplement: Figure S3 — Surface marker expression of ILC2 subsets. Lung c-kit+ (black line) and c-kit− (blue line) ILC2 subsets were analyzed for indicated surface markers between 10–12 d.p.i.. Isotype controls are represented as shaded histrograms. (TIF) [file ppat.1003615.s003.tif]

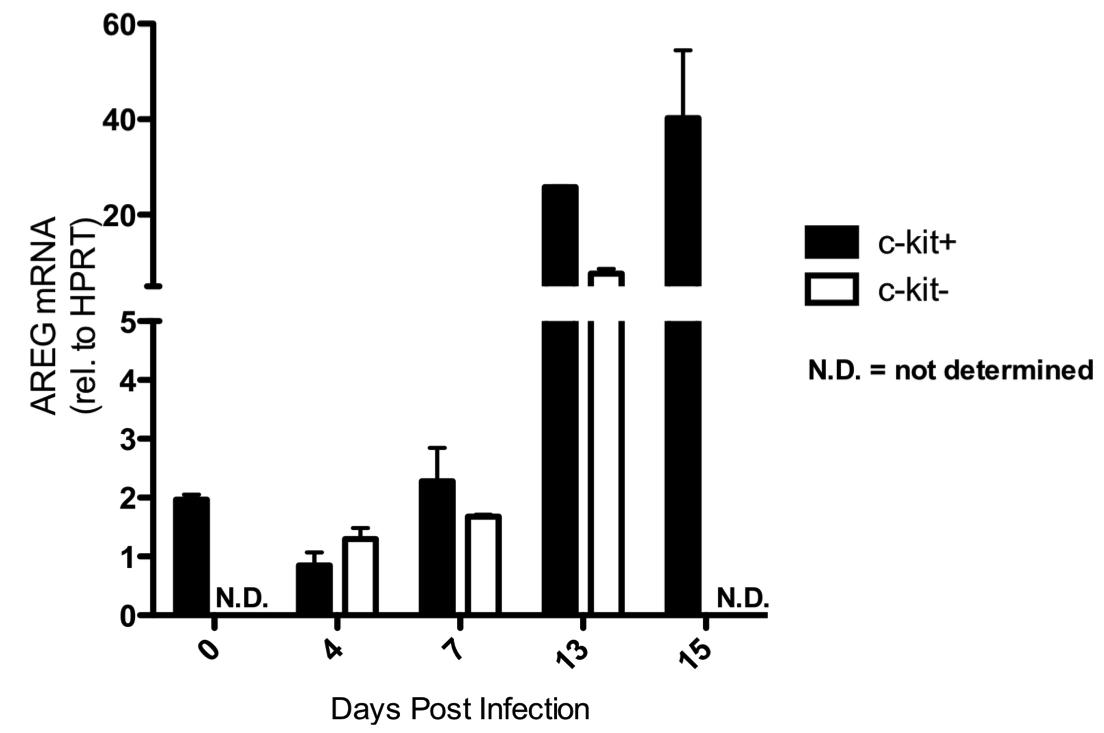

Supplement: Figure S4 — ILC2 express amphiregulin. ILC2 subsets were FACS sorted from the lung and analyzed for amphiregulin (areg) transcripts at indicated d.p.i.. N.D. = not determined. (TIF) [file ppat.1003615.s004.tif]

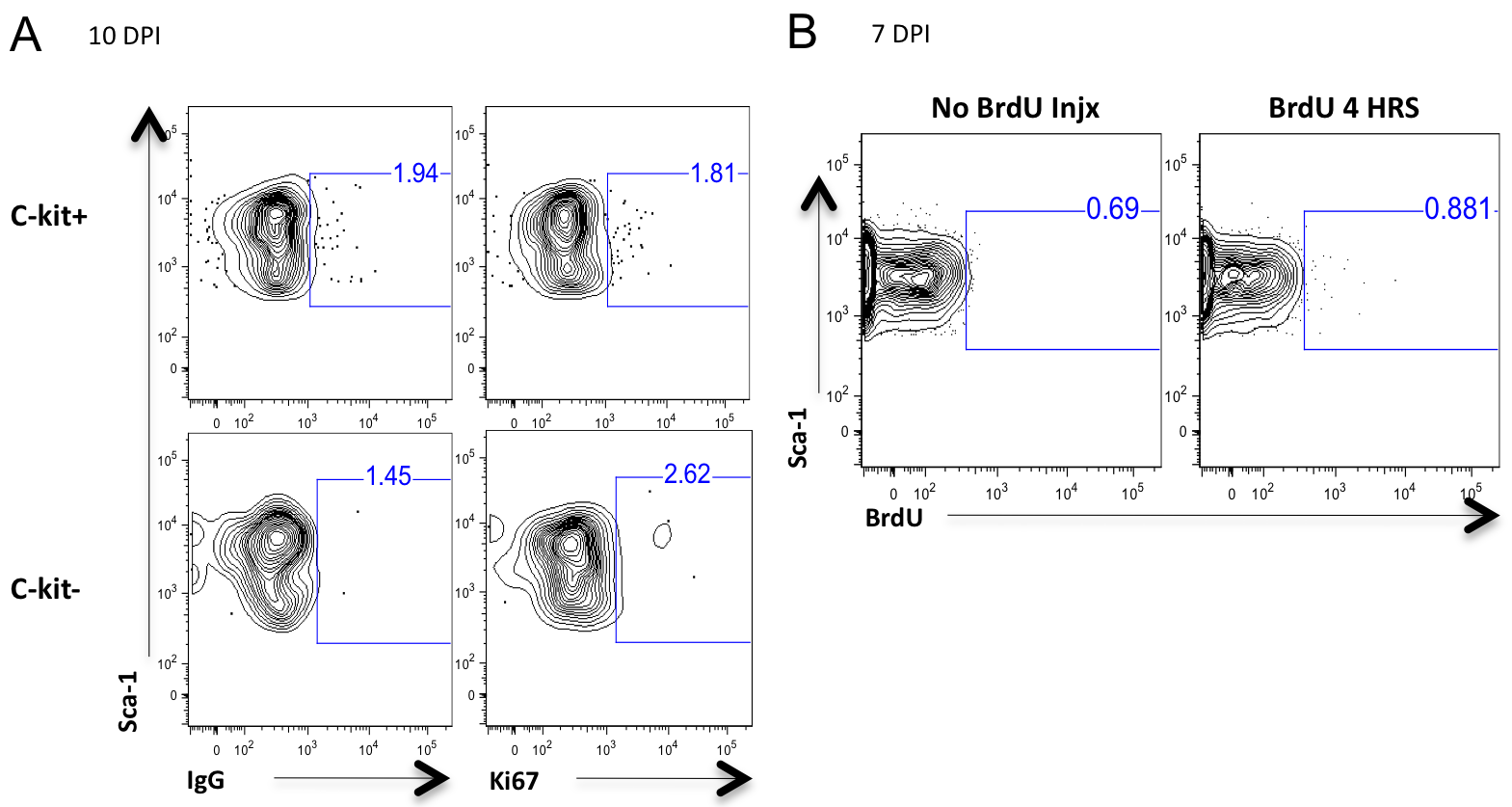

Supplement: Figure S5 — Group 2 innate lymphoid cells do not proliferate in the respiratory tract. (A) ILC2 subsets from 10 d.p.i. lung were intracellularly stained for the proliferation marker Ki67. (B) 7 d.p.i. mice were injected with BrdU 4 hours before harvesting the lungs and staining for BrdU. (TIF) [file ppat.1003615.s005.tif]

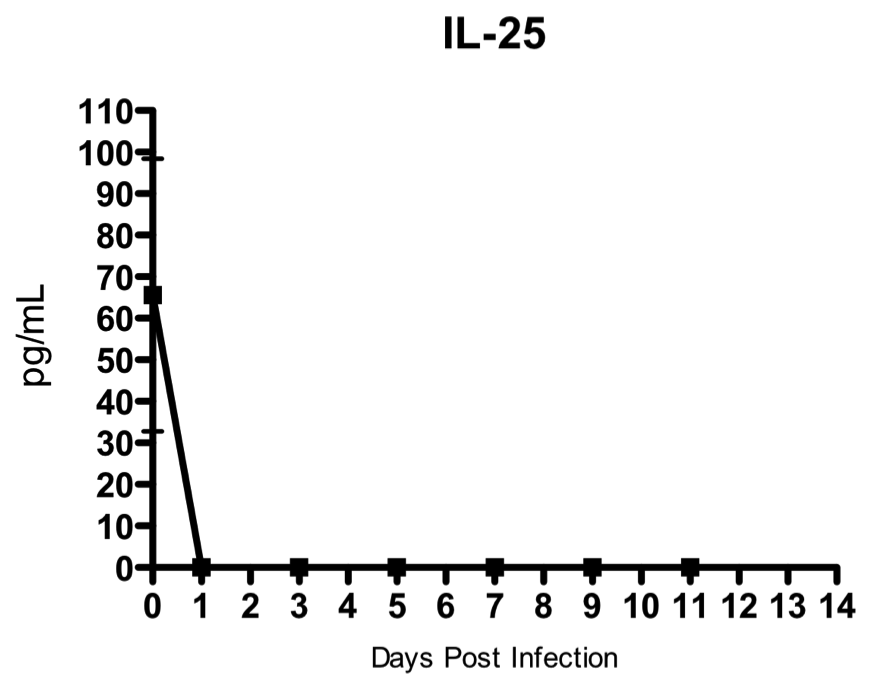

Supplement: Figure S6 — IL-25 is not detectable in the BAL during IAV infection. C57BL/6 mice were infected with PR8 and BAL fluid harvested at the indicated d.p.i.. Protein analyzed via Luminex. Limit of detection = .08 pg/ml. (TIF) [file ppat.1003615.s006.tif]

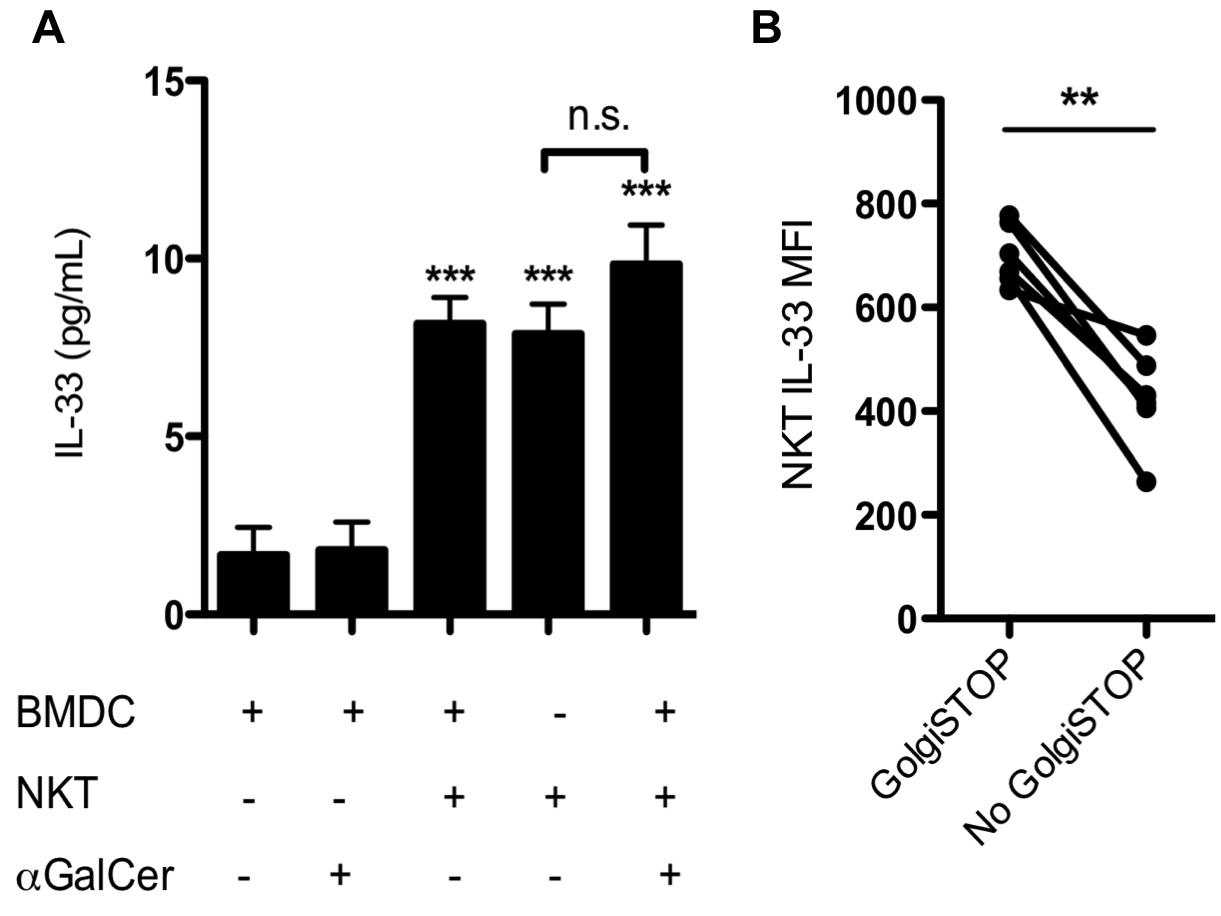

Supplement: Figure S7 — NKT cells secrete IL-33 protein. (A) NKT cells were MACS enriched from 7 d.p.i. lung cell suspensions (purity >92%) and cultured (2×10^5^ cells/well) with or without BMDC and/or 10 ng/ml αGalCer for 24–48 hours. Supernatants were analyzed for IL-33 via ELISA (Biolegend). (B) Intracellular IL-33 was analyzed in NKT cells from 12 d.p.i. lung cell suspensions cultured for 24 hours ex vivo with or without GolgiSTOP added for the last 4 hours of culture. n = 5–6 per group. Bars = +/− SEM. BMDC = bone marrow dendritic cell, n.s. non-significant. **p<.01, ***p<.001 (compared to BMDC alone). (TIF) [file ppat.1003615.s007.tif]

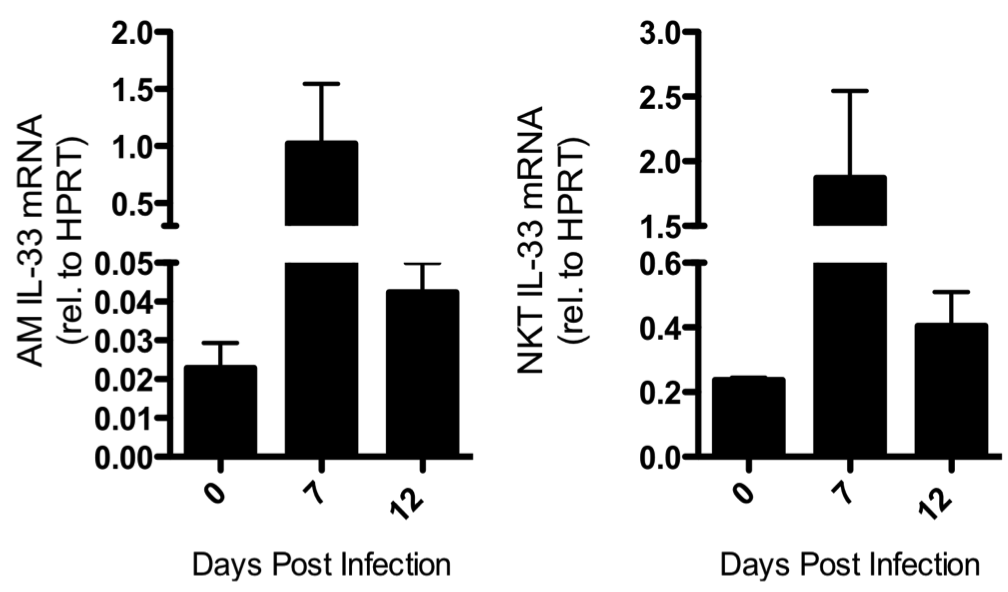

Supplement: Figure S8 — IAV infection induces IL-33 expression in alveolar macrophages and NKT cells. Alveolar macrophages (AM) and NKT cells were FACS sorted from the lung at indicated d.p.i. and analyzed for IL-33 transcript levels. Cell from n = 5–15 pooled lungs per day. (TIF) [file ppat.1003615.s008.tif]
